# Effects of Ensiling Duration on Fermentation Quality and Bacterial Communities of Fermented Total Mixed Ration Formulated with Corn Stalk and Broom Sorghum Straw

**DOI:** 10.3390/microorganisms14051052

**Published:** 2026-05-08

**Authors:** Jiayu Zhao, Yuqi Zou, Jian Bao, Pengbo Sun, Mingjian Liu, Baochao Bai, Xiangdong Liu, Yuxuan Wang, Shuai Du, Muqier Zhao

**Affiliations:** 1 College of Grassland Science, Inner Mongolia Agricultural University, Hohhot 010019, China; yuyo_66@163.com (J.Z.); 15548718972@163.com (Y.Z.); sunpengbo1107@163.com (P.S.); liumj_nm@163.com (M.L.); bbc66688@163.com (B.B.); 18847788933@163.com (X.L.); yx_wang7@163.com (Y.W.); dushuai_nm@sina.com (S.D.); 2 Key Laboratory of Forage Cultivation, Processing and High Efficient Utilization, Ministry of Agriculture, Hohhot 010019, China; 3 Key Laboratory of Grassland Resources, Ministry of Education, Hohhot 010019, China; 4 Inner Mongolia Academy of Agricultural and Animal Husbandry Sciences, Hohhot 010031, China; baojian19940203@163.com

**Keywords:** straw, fermented total mixed ration, nutritional quality, fermentation quality, in vitro digestibility, microbial community dynamics

## Abstract

Fermented total mixed ration (FTMR) is an effective approach to preserve low-quality crop residues. This study investigated the effects of ensiling duration (0, 3, 5, 7, 15 and 30 days) on nutritional dynamics, fermentation quality, in vitro digestibility, and bacterial communities of FTMR containing corn stalk and broom sorghum straw at 50% moisture. After 30 d of ensiling, FTMR had the highest crude protein content, lowest neutral detergent fiber content, peak lactic acid concentration, stable low pH, undetectable butyric acid, and maximum in vitro NDF digestibility. Bacterial alpha diversity declined significantly during ensiling, and the community was dominated by *Leuconostoc*, *Lactiplantibacillus*, and *Levilactobacillus*. These results clarify the microbial regulatory mechanism of mixed-straw FTMR fermentation and support the efficient utilization of crop residues in ruminant production.

## 1. Introduction

Fermented total mixed ration (FTMR) is prepared through the anaerobic ensiling process of conventional TMR. Driven primarily by lactic acid bacteria, this biological process can not only degrade complex macro-fibrous structures and anti-nutritional factors to improve digestion and absorption efficiency, but also enrich the feed with viable beneficial microorganisms and nutritionally active substances [1]. Compared to conventional TMR, which is susceptible to rapid aerobic deterioration, anaerobic fermentation can effectively maintain nutritional integrity and extend the storage period, thereby mitigating economic losses caused by spoilage [2]. Furthermore, FTMR is characterized by optimal moisture and good palatability. Liu et al. [3] found that feeding FTMR could increase feed intake and nutrient utilization, reduce fecal nitrogen excretion, improve economic benefits, and mitigate environmental pollution. Previous studies have also shown that feeding FTMR to ruminants can significantly increase feed intake and growth performance, while reducing disease morbidity [4,5].

As an abundant, cost-effective, and non-food competitive roughage resource, crop straws can not only meet the nutritional requirements of ruminants but also improve their production performance, yet they have not been fully utilized to date [6]. To overcome the inherent barriers to straw utilization, various feed processing technologies have been developed, including traditional ensiling (the focus of this study) and extrusion puffing [7]. Extrusion puffing can disrupt the fiber structure of straw to promote microbial attachment and proliferation, thereby enhancing its ruminal degradability and improving feed intake and utilization efficiency [8]. Broom sorghum is planted extensively across China [9]. Despite containing elevated levels of crude fiber, its straw is abundant in vital minerals—including nitrogen, potassium, calcium, and phosphorus—giving it considerable potential as a roughage source for herbivorous animals [10]. Furthermore, in vivo trials have demonstrated that utilizing microbially fermented straw for goat diets significantly enhances both their growth metrics and meat characteristics [11]. Corn straw can be used as a roughage source for herbivorous livestock [12,13]; its application value can be improved through methods such as straw ensiling, thereby reducing economic costs, alleviating feed shortages, and promoting the development of animal husbandry [14]. The precise nutritional formulation and sealed anaerobic fermentation process of FTMR can effectively reduce the risk of aerobic deterioration. By co-fermenting straw and other feed ingredients into FTMR, this technology not only overcomes the nutritional limitations of traditional single-forage diets, but also enriches the final feed with beneficial microorganisms and organic acids. Therefore, after ingestion by ruminants, FTMR can positively modulate the intestinal microbiota, thereby benefiting animal health and growth.

Consequently, this study aimed to formulate an FTMR based on a targeted mixed roughage of corn stalk and broom sorghum straw. Unlike previous studies that merely evaluated the final fermentation products of single substrates, this study highlights two core novelties. First, the co-fermentation of these two specific straws serves as a targeted nutritional strategy aimed at achieving nutrient complementarity. Second, this study systematically unveils the micro-ecological regulatory mechanisms across the entire ensiling continuum. We revealed the dynamic changes in nutritional value, fermentation quality, in vitro digestibility, and bacterial community succession at different fermentation durations (0, 3, 5, 7, 15, and 30 days). By coupling 16S rRNA sequencing with PICRUSt2 functional prediction, we mapped the temporal patterns of metabolic shifts, revealing the impact of microbial interactions on fermentation quality. The outcomes of this investigation are anticipated to yield a robust theoretical foundation and actionable recommendations for enhancing the utility and economic value of agricultural residues, thereby accelerating the broader technological implementation of straw resource utilization.

## 2. Materials and Methods

### 2.1. Fermented Total Mixed Ration Preparation

In late September 2024, raw materials including corn stalk and broom sorghum straw were collected from farmlands in Lindong Town, Bairin Left Banner, Chifeng City, Inner Mongolia Autonomous Region, China (43°57′ N, 119°21′ E), where these crops are planted each April. Following collection, the fresh forage was processed using mechanical chopping to achieve target lengths of approximately 2 to 3 cm. For the preparation of the FTMR, equal proportions (1:1 ratio) of the two chopped straws were combined to form the roughage base. This mixture was then uniformly integrated with a commercial concentrate sourced from Chaoyue Feed Co., Ltd. (Lindong, China). After the hydration level of the entire blend was adjusted to exactly 50%, portions of about 500 g of the well-mixed ration were placed into 35 cm × 40 cm polyethylene bags. These bags were immediately tightly closed via a commercial vacuum packaging device to ensure strict anaerobic conditions for fermentation.

A total of 18 vacuum-sealed bags (6 time points × 3 biological replicates) were prepared and stored at ambient temperature. At each designated ensiling time point (0, 3, 5, 7, 15, and 30 days), three separate bags (representing three independent biological replicates) were opened to investigate the temporal changes in nutritional value, silage characteristics, bacterial communities, and in vitro digestibility. No bag was opened multiple times in order to strictly maintain the anaerobic environment. The detailed feed composition of the FTMR is presented in Table 1.

### 2.2. Microbial Counts, Nutritional Quality and Fermentation Parameters

To extract the fermentation liquid from the fresh broom sorghum straw silage, 10 g of the FTMR mixture was blended with 90 mL of sterile aqueous solution and subjected to homogenization for 2 min. The acidity of the resulting extract was subsequently recorded utilizing a PHS-3C electrode pH meter (INESA Scientific Instrument Co., Ltd., Shanghai, China). Following the protocol established by Cheng et al. [15], the quantification of individual organic acids—specifically lactic acid (LA), acetic acid (AA), propionic acid (PA), and butyric acid (BA)—was conducted using an Agilent 1260 high-performance liquid chromatography (HPLC) system (Agilent Technologies, Inc., Waldbronn, Germany). This apparatus featured a refractive index detector and was fitted with a Carbomix^®^ H-NP5 analytical column (Sepax Technologies, Inc., Newark, DE, USA). The chromatographic operating conditions included a mobile phase of 2.5 mmol/L H_2_SO_4_ flowing at a rate of 0.5 mL/min, with the column temperature maintained strictly at 55 °C. Finally, the concentration of ammonia nitrogen (NH_3_-N) was evaluated by applying the phenol-sodium hypochlorite colorimetric assay [16].

To assess the chemical profile, three distinct biological replicates (representing individual vacuum bags) were examined for every fermentation time point. To calculate the dry matter (DM) fraction, aliquots of either the unensiled or fermented mixtures were placed into a forced-air oven maintained at 65 °C and dehydrated for a minimum of 72 h until their mass stabilized [17]. The Dumas combustion nitrogen technique was applied to quantify the crude protein (CP) levels [18], whereas the water-soluble carbohydrate (WSC) concentration was measured employing the anthrone colorimetric assay [19]. Following the analytical protocols outlined by Van Soest [20], the proportions of neutral detergent fiber (NDF) and acid detergent fiber (ADF) were evaluated utilizing an ANKOM A200i fiber analyzer unit (ANKOM Technology Corp., Fairport, NY, USA).

### 2.3. DNA Extraction, PCR and Sequencing

Total genomic DNA from the unensiled and ensiled FTMR blends (formulated with corn stalk and broom sorghum straw) was isolated employing a commercial E.Z.N.A.R DNA extraction kit (Omega Bio-tek, Norcross, GA, USA). This extraction process adhered to the protocols outlined by Huang [21]. The quality and quantity of the recovered DNA were subsequently evaluated via 1% agarose gel electrophoresis coupled with a NanoDrop 2000 UV-vis spectrophotometer (Thermo Fisher Scientific, Waltham, MA, USA). For the polymerase chain reaction (PCR) amplification targeting the V3-V4 hypervariable regions of the bacterial 16S rRNA gene, the specific primer pair 338F (5′-ACTCCTACGGGAGGCAGCAG-3′) and 806R (5′-GGACTACHVGGGTWTCTAAT-3′) was utilized. Following amplification, sequencing libraries were constructed utilizing the NEXTFLEX^®^ Rapid DNA-Seq Kit (Bioo Scientific, Austin, TX, USA). Finally, high-throughput sequencing was executed on an Illumina MiSeq PE300 platform provided by Shanghai Meiji Biomedical Technology Co., Ltd. (Shanghai, China).

### 2.4. Bacterial Community Sequencing Analysis

Raw paired-end sequencing reads were initially demultiplexed relying on their unique barcodes, followed by the trimming of adapter and barcode sequences. The paired reads were subsequently merged utilizing FLASH software (v1.2.8). To ensure data reliability, the raw sequences underwent stringent quality control via fqtrim (v0.94), yielding high-quality clean reads. Identification and removal of chimeric artifacts were executed with Vsearch (v2.3.4). The resulting curated datasets were then imported into the QIIME2 pipeline (v2019.7) for downstream bioinformatics workflows. Dereplication and generation of the feature table, along with representative sequences, were accomplished applying the Divisive Amplicon Denoising Algorithm 2 (DADA2).

Taxonomic classification of the representative sequences was assigned against the SILVA database (version 138), and feature abundances were normalized to facilitate accurate cross-sample comparisons. To assess variations in microbial richness and structure across different treatments, alpha and beta diversity metrics were computed utilizing the q2-diversity plugin within QIIME2 (v2019.7). Specifically, bacterial community shifts among groups were visualized through Principal Coordinate Analysis (PCoA) based on weighted UniFrac distances. Variations in the relative abundances of specific taxa between populations were statistically evaluated employing the Mann–Whitney U test.

To elucidate the interplay between the microbiota and silage characteristics, a Spearman correlation analysis was conducted to evaluate and visualize the associations linking key bacterial communities with fermentation parameters [22]. Furthermore, functional metabolic pathways of the bacterial communities within the FTMR were predicted utilizing the Phylogenetic Investigation of Communities by Reconstruction of Unobserved States 2 (PICRUSt2) tool, leveraging reference profiles from the KEGG database [23]. Finally, the Kruskal–Wallis H test was applied to detect significant shifts in the relative abundance of these predicted KEGG pathways across various ensiling periods, with statistical significance defined at *p* < 0.05.

### 2.5. Determination of In Vitro Digestibility

To replicate the gastrointestinal digestion process of ruminants, a DAISY II in vitro incubation apparatus was employed to assess both the 48 h in vitro dry matter digestibility (IVDMD) and in vitro neutral detergent fiber digestibility (IVNDFD) [24]. For this procedure, roughly 0.5 g of the fermented mixture was accurately weighed, placed into specialized filter bags, and securely sealed. Fresh rumen fluid, extracted from Simmental cattle prior to their morning meal, was strained through a four-layer cheesecloth to remove large particulates. Subsequently, 400 mL of the strained rumen liquor was combined with 1330 mL of Buffer A and 266 mL of Buffer B within a digestion vessel that had been pre-heated to 39 °C. Once the sealed sample bags were immersed in the mixture, the vessel was continuously purged with carbon dioxide (CO_2_) to ensure a strictly anaerobic environment. The jar was then tightly closed and transferred to the in vitro incubator, where it remained at a constant temperature of 39 °C for a duration of 48 h. Following the incubation period, the bags were retrieved from the jar and washed extensively with distilled water. Finally, the rinsed bags underwent drying and weighing to quantify the residual mass and the remaining undigested NDF fractions, providing the necessary data to compute the respective in vitro digestibility values.

The formulas are as follows:

IVDMD(%) = 100 ∗ (Mass of initial dry matter − Mass of undigested dry matter)/Mass of initial dry matter.

IVNDFD(%) = 100 ∗ (Initial NDF content − Undigested NDF content)/Initial NDF content.

Note: The composition of Buffer A included 10 g/L potassium dihydrogen phosphate, 0.5 g/L magnesium sulfate, 0.5 g/L sodium chloride, 0.1 g/L calcium chloride, and 0.5 g/L urea (analytical grade). Buffer B was prepared by dissolving 15 g/L sodium carbonate and 1 g/L sodium sulfide.

### 2.6. Data Analysis

Microsoft Excel 2021 was used for data collation. Alpha diversity, chemical composition and fermentation characteristics were analyzed using SAS ver. 9.2 (SAS Institute, 2007, Cary, NC, USA). Differences among means were analyzed using one-way analysis of variance (ANOVA) followed by Tukey’s HSD post hoc test, with *p* < 0.05 considered statistically significant. In this study, bacterial taxa with a relative abundance greater than 10% at the phylum or genus level in a specific treatment were defined as dominant.

## 3. Results

### 3.1. Raw Material Characteristics of Corn Stalk and Broom Sorghum Straw

The nutritional quality of the raw corn stalk and broom sorghum straw is presented in Table 2. The corn stalk exhibited a dry matter (DM) content of 76.58% on a fresh weight basis, with crude protein (CP), water-soluble carbohydrates (WSC), neutral detergent fiber (NDF), and acid detergent fiber (ADF) contents of 2.75%, 8.67%, 74.84%, and 44.95%, respectively. In contrast, the broom sorghum straw had a DM content of 90.80% on a fresh weight basis, alongside 2.80% CP, 5.67% WSC, 83.00% NDF, and 58.58% ADF.

### 3.2. Changes in the Nutritional Composition of FTMR Prepared from Corn Stalk and Broom Sorghum Straw

The dynamic changes in the nutritional composition of the FTMR prepared from corn stalk and broom sorghum straw across different ensiling durations are presented in Table 3. Ensiling duration had a significant effect on the dry matter (DM), crude protein (CP), water-soluble carbohydrates (WSC), and neutral detergent fiber (NDF) contents of the FTMR (*p* < 0.05). As fermentation progressed, the DM content exhibited a gradual decline (*p* < 0.05), whereas the CP content showed a progressive increase, reaching its maximum at 30 days of ensiling (*p* < 0.05). The WSC content initially decreased before subsequently stabilizing, with the highest concentration observed at day 0 (*p* < 0.05). Furthermore, the NDF content gradually declined with prolonged ensiling, dropping to its lowest level at day 30 (*p* < 0.05). Although the acid detergent fiber (ADF) content peaked at day 0, this temporal variation was not statistically significant (*p* > 0.05).

### 3.3. Changes in the Fermentation Quality of FTMR Prepared from Corn Stalk and Broom Sorghum Straw

The dynamic changes in the fermentation quality of the FTMR prepared from corn stalk and broom sorghum straw across different ensiling durations are presented in Table 4. Ensiling duration had a significant effect on the pH, lactic acid (LA), acetic acid (AA), and ammonia nitrogen (NH_3_-N) levels of the FTMR (*p* < 0.05). As fermentation progressed, the pH value exhibited a gradual decline (*p* < 0.05), whereas the LA and AA contents showed a progressive increase with prolonged ensiling. Specifically, the LA concentration peaked at 30 days (*p* < 0.05). In contrast, temporal variation had no significant effect on the propionic acid (PA) content, which reached its numerical maximum at 15 days (*p* > 0.05). Notably, butyric acid (BA) was not detected at any time point during the entire fermentation continuum.

### 3.4. Changes in the In Vitro Digestibility of FTMR Prepared from Corn Stalk and Broom Sorghum Straw

The dynamic changes in the in vitro digestibility of the FTMR prepared from corn stalk and broom sorghum straw at different ensiling durations are presented in Table 5. Ensiling duration only had a significant effect on the in vitro neutral detergent fiber digestibility (IVNDFD) of the FTMR (*p* < 0.05). With prolonged ensiling, the in vitro dry matter digestibility (IVDMD) exhibited a gradual upward trend, although this temporal variation was not statistically significant (*p* > 0.05). Conversely, the IVNDFD showed a progressive and significant increase over time, reaching its peak at 30 days of ensiling (*p* < 0.05).

### 3.5. Dynamics of Microbial Communities in FTMR Prepared with Corn Stalk and Broom Sorghum Straw

The changes in bacterial diversity indices of FTMR prepared with corn stalk and broom sorghum straw across different ensiling durations are presented in Table 6. The Shannon index reflects the diversity of the microbial community, with higher values indicating higher community diversity. The Ace and Chao indices are used to assess the species richness of the microbial community, with higher values representing greater species richness. The Simpson index is negatively correlated with community diversity, as larger index values correspond to lower community diversity. The Ace and Chao indices reached their maximum values at 0 d (*p* < 0.05), the Shannon index was also the highest at 0 d (*p* < 0.05), while the Simpson index peaked at 3 d with no significant difference among treatments (*p* > 0.05). The Good’s coverage of all sequenced samples exceeded 0.99.

**Figure 1:**
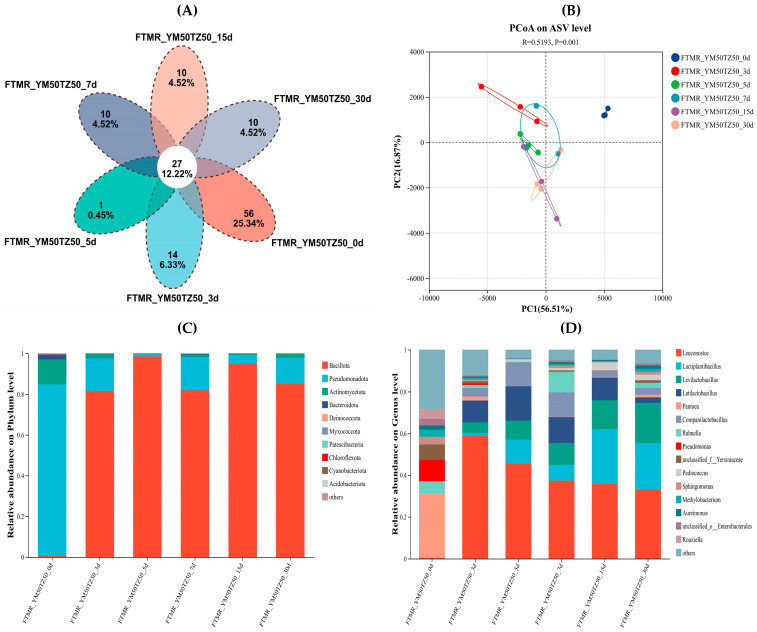
Bacterial community diversity and composition of FTMR prepared with corn stalk and broom sorghum straw across different ensiling durations. (**A**) Venn diagram of shared and unique ASVs. (**B**) PCoA of bacterial community structures. (**C**) Relative abundance of the top 10 bacterial phyla. (**D**) Relative abundance of the top 15 bacterial genera. Note: FTMR, fermented total mixed ration; YM50TZ50 represents a 50:50 (*w*/*w*) ratio of corn stalk to broom sorghum straw in the FTMR.

Venn analysis of bacteria at ASV level of FTMR forage prepared with corn stalk and broom sorghum straw under different ensiling durations is shown in Figure 1A. Among them, the number of shared ASVs in each group under different ensiling durations is 27, and the number of unique ASVs at 0 d is the highest, which is 56. The number of ASVs in samples under different ensiling durations decreased to 14, 1 and 10.

The PCoA analysis of bacterial communities in FTMR prepared with corn stalk and broom sorghum straw across different ensiling durations is shown in Figure 1B. Distinct clustering of bacterial communities was observed among the six treatment groups, indicating that the bacterial community structure was reshaped during ensiling, and that ensiling duration had a significant effect on the β-diversity of the microbial community. The variance explained by PC1 and PC2 was 56.51% and 16.87%, respectively.

Succession of bacterial communities at phylum and genus levels is presented in Figure 1C,D. At the phylum level (Figure 1C), Pseudomonadota and Actinomycetota had the highest relative abundance at 0 d. After ensiling, Bacillota exhibited the highest relative abundance and became the dominant phylum, followed by Pseudomonadota, while the relative abundance of other bacterial phyla remained at a low level. At the genus level (Figure 1D), *Pantoea* was the dominant genus at 0 d. *Leuconostoc* was the dominant genera at 3 d, while *Leuconostoc*, *Lactiplantibacillus*, *Levilactobacillus* and *Latilactobacillus* were the dominant genera during the late stage of ensiling.

**Figure 2:**
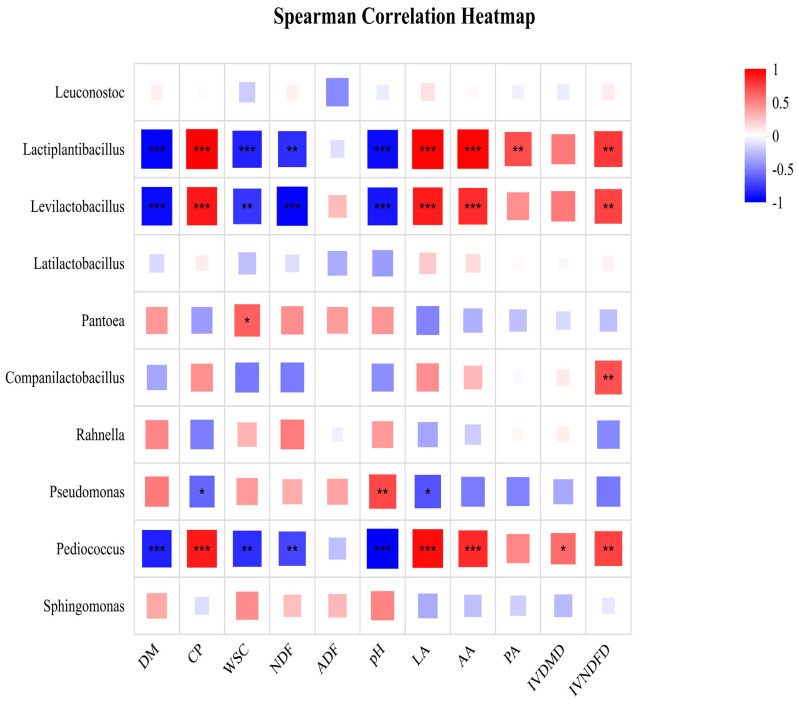
Spearman correlation heatmap between bacterial communities and fermentation characteristics in FTMR prepared with corn stalk and broom sorghum straw. red indicates positive correlation, and blue indicates negative correlation. The significance level was: * *p* < 0.05; ** 0.001 < *p* < 0.01; *** *p* < 0.001.

The correlation between the nutritional and fermentation quality of FTMR prepared with corn stalk and broom sorghum straw and bacterial genera is shown in Figure 2. Both *Lactiplantibacillus* and *Levilactobacillus* exhibited significant negative correlations with DM, WSC, NDF, and pH, while showing significant positive associations with CP, LA, AA, and IVNDFD (*p* < 0.05). A significant positive correlation was observed between *Pantoea* and WSC (*p* < 0.05), and similarly, *Companilactobacillus* was positively related to IVNDFD (*p* < 0.05). In contrast, *Pseudomonas* displayed significant negative linkages with CP and LA, but was significantly positively associated with pH (*p* < 0.05). Finally, *Pediococcus* demonstrated significant negative correlations with DM, WSC, NDF, and pH, whereas it was significantly positive correlated with CP, LA, AA, IVDMD, and IVNDFD (*p* < 0.05).

### 3.6. Metabolic Pathway Prediction of FTMR Prepared with Corn Stalk and Broom Sorghum Straw at Three Levels

**Figure 3:**
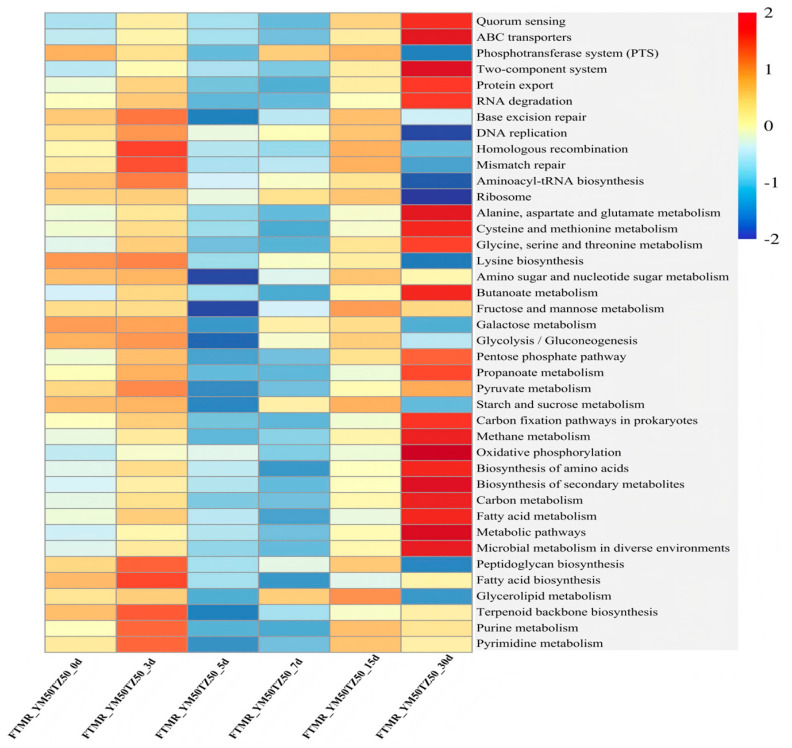
Heatmap of predicted core tertiary functional pathways (KEGG Level 3) of the bacterial community in FTMR formulated with corn stalk and broom sorghum straw across different ensiling stages. Note: FTMR, fermented total mixed ration; YM50TZ50 represents a 50:50 (*w*/*w*) ratio of corn stalk to broom sorghum straw in the FTMR.

**Figure A1:**
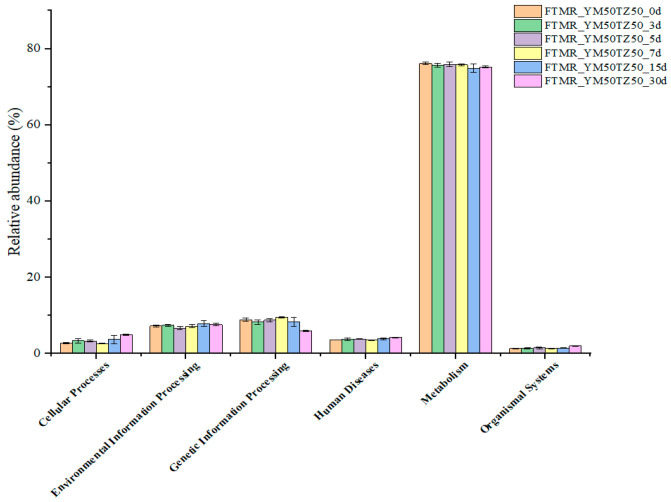
Predicted functional profiles of the bacterial community in FTMR formulated with corn stalk and broom sorghum straw at the primary pathway level (KEGG Level 1). Note: FTMR, fermented total mixed ration; YM50TZ50 represents a 50:50 (*w*/*w*) ratio of corn stalk to broom sorghum straw in the FTMR.

The 16S rRNA gene function prediction analysis of FTMR prepared with corn stalk and broom sorghum straw is shown in Figure 3. In the following analysis, all temporal differences in functional pathways described as ‘significant’ or ‘significantly enriched’ indicate statistical significance verified by the Kruskal–Wallis H test (*p* < 0.05). At the primary pathway level (KEGG Level 1, Figure A1), the overall functional profiles were largely consistent across all samples, with Metabolism being the most highly enriched category relative to other primary pathways. Significant temporal differences were observed in core primary pathways: Genetic Information Processing was significantly enriched at the mid-ensiling stage (day 7) compared with both the initial (day 0) and late mature (day 30) stages, while Environmental Information Processing and Cellular Processes were significantly more abundant at day 30 than at day 0.

**Figure A2:**
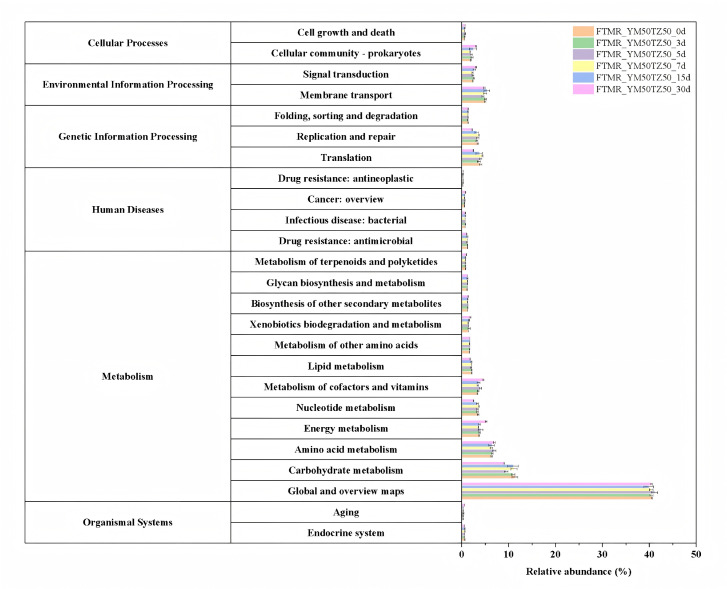
Predicted functional profiles of the bacterial community in FTMR formulated with corn stalk and broom sorghum straw at the secondary pathway level (KEGG Level 2). Note: FTMR, fermented total mixed ration; KEGG, Kyoto Encyclopedia of Genes and Genomes. YM50TZ50 represents a 50:50 (*w*/*w*) ratio of corn stalk to broom sorghum straw in the FTMR.

Consistent with the temporal shifts observed at the primary pathway level, global and overview maps, carbohydrate metabolism, and amino acid metabolism constituted the three major secondary pathways (KEGG Level 2, Figure A2) within the Metabolism category. Carbohydrate metabolism was most enriched at the initial stage (day 0) compared with all subsequent ensiling stages. The mid-ensiling stage (day 7) saw significant enrichment of proliferation-related pathways, including translation and replication and repair. At the late mature stage (day 30), pathways related to amino acid metabolism, energy metabolism, membrane transport, and signal transduction were significantly enriched.

At KEGG Level 3 (Figure 3), core functional pathways showed distinct temporal succession, with significantly higher relative abundances than other metabolic pathways. At day 0, pathways for readily fermentable carbohydrate utilization (galactose metabolism, glycolysis/gluconeogenesis, and starch and sucrose metabolism) were most enriched. At the early ensiling stage (day 3), pathways associated with bacterial growth and cellular proliferation (DNA replication, homologous recombination, and peptidoglycan biosynthesis) were significantly more abundant than at day 0. At day 30, pathways related to environmental sensing and substrate transport (quorum sensing, ABC transporters, and the two-component system), alongside key amino acid metabolism, dominated the functional profile.

## 4. Discussion

Ensiling duration has a significant effect on the dynamic changes in nutritional components in FTMR prepared with corn stalk and broom sorghum straw, and the ensiling process can significantly improve the nutritional value of feed through microbial metabolism. In addition, fermented feed can effectively replace the partial use of antibiotics, as its beneficial bacterial communities such as lactic acid bacteria can inhibit the proliferation of pathogenic bacteria and reduce the incidence of animal diseases [4,5]. In-depth analysis of the variation pattern of its nutritional components is of great value for promoting green livestock farming, ensuring food safety, and facilitating the sustainable development of animal husbandry. In this study, DM content showed a downward trend with the extension of ensiling duration, which may be related to the decomposition of organic matter by microorganisms during the ensiling process [25]. CP content reached its peak at 30 d, which was associated with the transformation of nitrogen sources driven by enhanced microbial metabolic activity. This finding is consistent with the study of Yang et al. [26], who reported that nitrogen compounds peaked at the end of the ensiling period, reflecting intense microbial metabolic activity, namely the conversion of non-protein nitrogen into microbial cell components, which further affected the overall protein profile of fermented TMR. After the initial active phase, the residual WSC content reached a plateau, indicating a state of metabolic equilibrium where the inhibition by low pH restricted further extensive sugar degradation by both lactic acid bacteria and remaining spoilage microflora [27]. The continuous decrease in NDF content reflects the gradual degradation of cellulose and hemicellulose during the ensiling process, which is consistent with the findings of G. Bretschneider et al. [28]. The above results indicate that an ensiling cycle of 30 d exerts the optimal effect on increasing CP content and reducing NDF content.

The results of this study showed that ensiling duration had a significant effect on the dynamic changes in fermentation quality of FTMR prepared with corn stalk and broom sorghum straw. Fermentation quality is the core guarantee of feed nutritional value and feeding safety. In this study, with the extension of ensiling duration, pH showed a significant downward trend, which may be closely related to the rapid proliferation and acid production metabolism of lactic acid bacteria [29]. In the early stage of fermentation, lactic acid bacteria preferentially used WSC to produce LA, leading to a rapid decrease in pH and inhibiting the activity of harmful microorganisms at the same time. LA content reached the peak at 30 d, indicating that the metabolic activity of lactic acid bacteria was in the dominant stage at this time, and the carbon source supply and microbial demand reached a dynamic balance. The continuous accumulation of AA indicated that the degradation of fiber structure in the middle and late ensiling stages released more fermentable substrates, which enhanced the metabolic activity of heterofermentative lactic acid bacteria and further promoted AA production [25]. PA content reached the highest value at 15 d, but there was no significant difference. In addition, BA was not detected in all treatments, indicating that the fermentation system effectively inhibited the growth of spoilage bacteria such as Clostridium, and further verified the inhibitory effect of acidic environment dominated by lactic acid bacteria on spoilage metabolism [30]. The above results show that the 30 d fermentation cycle has the best performance in increasing LA content and reducing pH.

The IVNDFD of the FTMR increased significantly with increasing ensiling duration, peaking at 30 d, which effectively improved the fiber utilization efficiency of the ration. This is consistent with the research results of Menke et al. [31] that the staged degradation of substrates during in vitro digestion is closely related to the dynamic succession of microbial communities. With the extension of ensiling duration, the activity of fibrolytic bacteria was enhanced, which promoted the hydrolysis of cellulose and hemicellulose.

The diversity, structure and function of microbial communities have long been a research hotspot in the field of feed science [32]. Monitoring the diversity of microbial communities is one of the key methods to investigate the effects of microorganisms on the ensiling process [33]. The results of this study showed that the bacterial diversity and richness of FTMR prepared with corn stalk and broom sorghum straw peaked at the early stage of ensiling, with the Ace and Chao indices significantly higher than those at other time points, indicating that the microbial community in the raw material had high species richness. With the extension of ensiling duration, the Shannon index decreased gradually, and the Simpson index reached the maximum value at 3 d, indicating that the diversity of the microbial community was inhibited by the competitive proliferation of dominant bacterial communities. The sequencing coverage of all samples in this study exceeded 0.99, which further verified the reliability of the sequencing data and indicated that the changes in the microbial community were mainly derived from ecological selection during the ensiling process. The species richness and diversity were the highest at 0 d, and both decreased after ensiling. This may be attributed to the formation of an anaerobic environment with the prolongation of ensiling duration, where lactic acid bacteria became the dominant flora. Lactic acid bacteria utilize WSC to produce LA, which rapidly creates an acidic environment to inhibit the growth and reproduction of other spoilage microorganisms [34].

The composition, structure and function of microbial communities are the core factors of the ensiling process. As the ensiling process relies on the interactions of multiple bacterial taxa, the structure of the bacterial community directly affects fermentation quality [35]. For FTMR prepared with corn stalk and broom sorghum straw, *Pseudomonadota* and *Actinomycetota* were the dominant phyla at the initial stage. At the phylum level, the relative abundance of *Pseudomonadota* decreased after ensiling and was gradually replaced by Bacillota. At the genus level, the results showed that there were significant differences in the bacterial community composition of FTMR prepared with corn stalk and broom sorghum straw at the early stage of ensiling. However, with the extension of ensiling duration, *Leuconostoc* and *Latilactobacillus* gradually became the dominant flora at 3 d, and *Leuconostoc*, *Lactiplantibacillus* and *Levilactobacillus* were the dominant genera during the late ensiling stage. In terms of bacterial community characteristics, lactic acid bacteria were consistently the dominant flora throughout the ensiling process of FTMR prepared with corn stalk and broom sorghum straw, indicating a favorable fermentation effect.

Correlation analysis of bacterial communities at the genus level was performed to reveal the direct or indirect relationships between microbial community structure, the ensiling process and nutrient preservation efficiency, which serves as a critical link between microbial ecology and the nutritional value of feed. In this study, the correlations between different bacterial genera and the nutritional and fermentation quality of FTMR prepared with corn stalk and broom sorghum straw revealed the fine regulatory mechanism of microbial communities on the ensiling process. *Lactiplantibacillus*, *Levilactobacillus* and *Pediococcus* were significantly positively correlated with NDF, indicating that *Lactiplantibacillus*, *Levilactobacillus* and *Pediococcus* may retard the rate of fiber degradation by secreting specific enzymes, thereby promoting its own growth [36,37,38]. As the core lactic acid bacteria in the ensiling process, *Lactiplantibacillus* was significantly positively correlated with DM, WSC, NDF and pH, and negatively correlated with CP, LA, AA and other indicators in this study. This indicated that when WSC was depleted in the middle and late ensiling stages, the metabolic activity of *Lactiplantibacillus* was limited by substrate availability. Consequently, its relative abundance was positively correlated with undegraded fiber components and residual DM, while showing an antagonistic relationship with the accumulation of CP, LA, and AA. Similarly, *Levilactobacillus* and *Pediococcus* were positively correlated with DM, WSC, NDF and pH, and negatively correlated with CP, LA and other indicators, which collectively reflected the synergistic restrictive effect of these lactic acid bacteria genera on substrate utilization in the ensiling system [39].

The functional profiles of the bacterial community, encompassing metabolic pathways and genetic information, were predicted from the 16S rRNA sequencing data utilizing the PICRUSt2 pipeline in conjunction with the KEGG database [40]. In the fermented total mixed ration (FTMR) formulated with corn stalk and broom sorghum straw, Metabolism emerged as the overwhelmingly dominant primary pathway (KEGG Level 1, Figure A1) throughout the entire ensiling process, with a stable relative abundance maintained at 73.9–76.2%. This indicates that ensiling fermentation is fundamentally driven by robust microbial metabolic activities, wherein microorganisms utilize available substrates in raw materials to sustain growth and reproduction, while converting organic matter into functional metabolites that shape the final fermentation quality [41]. At KEGG Level 2 (Figure A2), distinct temporal dynamics were observed across ensiling durations in core metabolic pathways, including global and overview maps, carbohydrate metabolism, amino acid metabolism, nucleotide metabolism, energy metabolism, and metabolism of cofactors and vitamins, as well as non-metabolic pathways closely related to microbial proliferation and environmental adaptation (translation, replication and repair, membrane transport, signal transduction). Previous studies have underscored that these core functional pathways are directly coupled with the succession of dominant bacterial communities, and play a pivotal role in determining the fermentation quality and nutritional value of the final silage product [42].

To further elucidate the stage-specific metabolic shifts during ensiling, tertiary pathways (KEGG Level 3, Figure 3) were analyzed in detail. At the initial stage of ensiling (day 0), the pronounced enrichment of carbohydrate utilization pathways (including galactose metabolism, glycolysis/gluconeogenesis, and starch and sucrose metabolism) reflects the rapid fermentation of water-soluble carbohydrates by lactic acid bacteria (LAB). This rapid carbohydrate catabolism drives robust lactic acid production and a sharp decline in system pH, which is critical for inhibiting spoilage microorganisms and preserving the nutritional components of the forage. Subsequently, at the early to mid-ensiling stage (days 3–7), pathways related to bacterial proliferation (e.g., DNA replication, homologous recombination, and peptidoglycan biosynthesis) were significantly enriched, which corresponded to the rapid growth of LAB. As fermentation proceeded (days 5–15), the continuous decline in pH and the depletion of easily fermentable substrates drove a successional shift in the bacterial community, leading to a relative decrease in proliferation-related metabolic activities. By the mature stage (day 30), to maintain cellular viability in the highly acidic and nutrient-limited environment, the dominant LAB dynamically upregulated specific adaptive pathways, including environmental sensing and substrate transport (e.g., two-component system, ABC transporters, and quorum sensing). It is worth noting that dominant lactic acid bacteria at this stage, such as *Lactiplantibacillus*, possess extensive genomic repertoires for these specific pathways. Under severe substrate limitation and low-pH stress, these homofermentative bacteria heavily rely on ABC transporters to scavenge residual nutrients (e.g., peptides and amino acids) and utilize two-component systems and quorum sensing to regulate acid tolerance responses and maintain cellular viability [43,44].

## 5. Conclusions

This study systematically analyzed the effects of different ensiling durations on the nutritional components, fermentation quality, in vitro digestibility, and microbial communities of FTMR prepared with corn stalk and broom sorghum straw. The results showed that 30 d was the optimal ensiling cycle for this FTMR: under this cycle, the CP content reached its peak, while the NDF content decreased significantly, which effectively improved the nutritional value of the ration; the LA content reached the maximum level, the pH of the fermentation system was stabilized within the optimal range, the growth and reproduction of harmful spoilage bacteria were effectively inhibited, and excellent fermentation quality was achieved; the IVNDFD peaked at this cycle, indicating that the utilization efficiency of dietary fiber components reached the optimal level. The dynamic analysis of microbial communities showed that lactic acid bacteria dominated the entire ensiling process in the middle and late ensiling stages. The 30 d ensiling cycle can effectively improve production efficiency while comprehensively ensuring the nutritional quality and digestibility of the feed, and reducing the risk of feed spoilage.

## Figures and Tables

**Table 1:** Ingredients of the fermented total mixed ration.

Ingredients	Mixing Ratios (%)
Corn stalk	20.0
Broom sorghum straw	20.0
Cornmeal	60.0
43% Extruded soybean meal	
DDGS	
Palm fat powder	
Corn fiber	
Dicalcium phosphate	
Limestone powder	
Corn gluten meal	
Sodium bicarbonate	
Salt	
Molasses	

**Table 2:** Nutritional composition of corn stalk and broom sorghum straw.

	DM (%FW)	CP (%DM)	WSC (%DM)	NDF (%DM)	ADF (%DM)
Corn stalk	76.58 ± 1.48	2.75 ± 0.52	8.67 ± 0.01	74.84 ± 0.02	44.95 ± 0.01
Broom sorghum straw	90.80 ± 1.56	2.80 ± 0.33	5.67 ± 0.01	83.00 ± 0.03	58.58 ± 0.03

FW, fresh weight; DM, dry matter; CP, crude protein; NDF, neutral detergent fiber; ADF, acid detergent fiber; WSC, water soluble carbohydrates.

**Table 3:** Dynamic changes in the nutritional composition of FTMR at different ensiling durations.

Time	DM (%FW)	CP (%DM)	WSC (%DM)	NDF (%DM)	ADF (%DM)
0	52.39 ± 0.27 ^a^	15.31 ± 0.08 ^d^	6.67 ± 0.58 ^a^	52.04 ± 1.01 ^a^	35.42 ± 2.77 ^a^
3	52.16 ± 0.20 ^ab^	15.31 ± 0.02 ^d^	5.19 ± 0.36 ^b^	51.46 ± 1.97 ^ab^	34.15 ± 1.73 ^a^
5	52.04 ± 0.12 ^b^	15.44 ± 0.05 ^bcd^	4.80 ± 0.19 ^b^	49.87 ± 0.74 ^ab^	33.37 ± 2.29 ^a^
7	51.92 ± 0.09 ^abc^	15.54 ± 0.09 ^bc^	4.31 ± 0.16 ^b^	48.81 ± 1.22 ^abc^	33.13 ± 1.04 ^a^
15	51.31 ± 0.40 ^bc^	15.69 ± 0.12 ^b^	4.43 ± 0.12 ^b^	46.61 ± 3.75 ^bc^	34.38 ± 2.54 ^a^
30	51.21 ± 0.56 c	15.93 ± 0.12 ^a^	4.34 ± 0.62 ^b^	44.53 ± 1.49 ^c^	35.06 ± 1.40 ^a^
*p*-value	**	***	***	**	0.7180

DM, dry matter; CP, crude protein; NDF, neutral detergent fiber; ADF, acid detergent fiber; WSC, water soluble carbohydrates. ** 0.001 < *p* < 0.01; *** *p* < 0.001. Different lowercase superscript letters in the same column indicate significant differences among different ensiling durations (*p* < 0.05).

**Table 4:** Dynamic changes in the fermentation quality of FTMR at different ensiling durations.

Time	pH	LA (g/kg FM)	AA (g/kg FM)	PA (g/kg FM)	BA (g/kg FM)	NH_3_-N (g/kg FM)
0	6.14 ± 0.15 ^a^	0.48 ± 0.01 ^e^	0.03 ± 0.01 ^c^	0.01 ± 0.01 ^a^	ND	0.17 ± 0.01 ^b^
3	5.04 ± 0.06 ^b^	0.59 ± 0.02 ^de^	0.04 ± 0.01 ^bc^	0.01 ± 0.00 ^a^	ND	0.15 ± 0.00 ^ab^
5	4.59 ± 0.07 ^c^	0.75 ± 0.09 ^cd^	0.05 ± 0.02 ^bc^	0.02 ± 0.01 ^a^	ND	0.23 ± 0.03 ^a^
7	4.47 ± 0.10 ^cd^	0.94 ± 0.03 ^bc^	0.07 ± 0.00 ^ab^	0.02 ± 0.00 ^a^	ND	0.14 ± 0.02 ^ab^
15	4.40 ± 0.09 ^cd^	1.07 ± 0.15 ^ab^	0.08 ± 0.01 ^a^	0.03 ± 0.02 ^a^	ND	0.11 ± 0.01 ^c^
30	4.34 ± 0.03 ^e^	1.24 ± 0.16 ^a^	0.09 ± 0.00 ^a^	0.02 ± 0.01 ^a^	ND	0.16 ± 0.01 ^ab^
*p*-value	***	***	***	0.1580	/	***

LA, lactic acid; AA, acetic acid; PA, propionic acid; BA, butyric acid; NH_3_-N, ammonia nitrogen. ND, not detected. *** *p* < 0.001. Different lowercase superscript letters in the same column indicate significant differences among different ensiling durations (*p* < 0.05).

**Table 5:** Dynamic changes in in vitro digestibility at different ensiling durations.

Time	IVDMD (%DM)	IVNDFD (%DM)
0	30.92 ± 3.34	43.26 ± 2.69 ^b^
3	31.35 ± 1.48	46.34 ± 1.90 ^b^
5	31.45 ± 1.97	46.80 ± 4.67 ^b^
7	32.94 ± 2.67	47.39 ± 4.09 ^b^
15	33.82 ± 1.82	48.23 ± 2.38 ^ab^
30	35.70 ± 3.17	54.90 ± 0.80 ^a^
*p*-value	0.2310	*

IVDMD, in vitro dry matter digestibility; IVNDFD, in vitro neutral detergent fiber digestibility. * *p* < 0.05. Different lowercase superscript letters in the same column indicate significant differences among different ensiling durations (*p* < 0.05).

**Table 6:** Dynamic changes in bacterial alpha diversity under different ensiling durations.

Time	Ace	Chao	Shannon	Simpson	Coverage
0	236.07 ± 46.92 ^a^	231.28 ± 45.32 ^a^	3.27 ± 0.35 ^a^	0.12 ± 0.05 ^a^	0.99 ± 0.00
3	148.14 ± 32.10 ^ab^	146.42 ± 31.48 ^ab^	2.16 ± 0.67 ^ab^	0.31 ± 0.23 ^a^	0.99 ± 0.00
5	65.43 ± 5.36 ^b^	64.55 ± 5.45 ^b^	1.95 ± 0.11 ^b^	0.24 ± 0.04 ^a^	0.99 ± 0.00
7	127.84 ± 40.12 ^b^	123.42 ± 40.87 ^b^	2.19 ± 0.23 ^ab^	0.21 ± 0.03 ^a^	0.99 ± 0.00
15	126.13 ± 28.88 ^b^	121.84 ± 26.43 ^b^	2.17 ± 0.10 ^ab^	0.21 ± 0.04 ^a^	0.99 ± 0.00
30	124.19 ± 58.83 ^b^	123.16 ± 58.49 ^b^	2.56 ± 0.68 ^ab^	0.17 ± 0.07 ^a^	0.99 ± 0.00
*p*-value	**	**	*	0.3720	—

* *p* < 0.05; ** 0.001 < *p* < 0.01. Different lowercase superscript letters in the same column indicate significant differences among different ensiling durations (*p* < 0.05).

## Data Availability

The original data presented in the study are openly available in NCBI under study record number PRJNA1452755.
